# Combination Between Biomarkers and Echocardiographic Data for Prediction of Left Ventricular Reverse Remodelling in Cardiac Resynchronization Therapy

**DOI:** 10.3390/jcm14103496

**Published:** 2025-05-16

**Authors:** Matteo Beltrami, Alessandro Galluzzo, Giacomo Bonacchi, Luca Checchi, Giuseppe Ricciardi, Laura Perrotta, Manuel Garofalo, Alessandro Paoletti Perini, Alessio Mattesini, Paolo Pieragnoli, Alberto Palazzuoli

**Affiliations:** 1 Arrhythmia and Electrophysiology Unit, Careggi University Hospital, 50134 Florence, Italy; checchil@aou-careggi.toscana.it (L.C.); ricciardig@aou-careggi.toscana.it (G.R.); perrottal@aou-careggi.toscana.it (L.P.); manuel.garofalo@unifi.it (M.G.); pieragnolip@aou-careggi.toscana.it (P.P.); 2 Cardiology Unit, Rivoli Hospital, 10098 Turin, Italy; alegallu@yahoo.it; 3 Cardiomyopathy Unit, Careggi University Hospital, 50134 Florence, Italy; bonacchig@aou-careggi.toscana.it; 4 School of Human Health Sciences, University of Florence, 50134 Florence, Italy; 5 Cardiology and Electrophysiology Unit, Santa Maria Nuova Hospital, 50134 Florence, Italy; 6 Department of Structural Interventional Cardiology, Careggi University Hospital, 50134 Florence, Italy; 7 Cardiovascular Diseases Unit, Cardio Thoracic and Vascular Department, Le Scotte Hospital, University of Siena, 53100 Siena, Italy; palazzuoli2@unisi.it

**Keywords:** cardiac resynchronization therapy, galectin-3, soluble suppression of tumorigenicity-2, heart failure with reduced ejection fraction, biomarkers

## Abstract

**Purpose**: Although biomarkers of myocardial fibrosis and inflammation have been proposed as potential modulators of response to cardiac resynchronization therapy (CRT), their clinical utility and interaction with echocardiographic parameters remain incompletely understood. This study aims to assess the dynamic changes in these biomarkers, their relationship with echocardiographic variables, and their association with structural response to CRT. **Methods**: We retrospectively evaluated 86 consecutive patients referred for CRT with symptomatic heart failure, left ventricular (LV) ejection fraction ≤ 35%, QRS width ≥ 130 ms and LBBB morphology. We measured sST-2, Gal-3, NTpro-BNP and eGFR at baseline and after 1 year of CRT. An echocardiographic reduction of LV end-systolic volume ≥ 15% was used to define a patient as a responder to CRT. **Results**: The mean baseline and follow-up values of Gal-3 (responders: 24.1 [16.8;32] ng/mL, non-responders: 30 [20;39.3] ng/mL, *p* = 0.03) and sST2 (responders: 28.5 [20;36] ng/mL, non-responders: 34.5 [25;37.7] ng/mL, *p* = 0.03) were lower in responders than non-responders. Responders showed a significant reduction between baseline and follow-up values of ΔGal-3 (−12.1% vs. −2.5%, *p* = 0.04), ΔsST2 (−30.8% vs. 2.2%, *p* < 0.001), ΔNT-proBNP (−16.4% vs. 5.2, *p* = 0.04) and ΔeGFR (6.7 ± 24.3% vs. -6.3 ± 27.9%, *p* = 0.03). At the multivariate analyses, baseline Gal-3 [cut-off: 38.5 ng/mL, AUC: 0.63, *p* = 0.03, (OR 7.13 [1.12;45.41], *p* = 0.03), together with TAPSE > 17.5 mm (OR 10.86 [3.15;37.44], *p* < 0.001) significantly correlated with the structural response to CRT in several prediction models. Among echocardiographic parameters, TAPSE remained the strongest predictive factor of positive response to CRT at the univariate and multivariate analyses. **Conclusions**: In patients with heart failure and reduced ejection fraction undergoing CRT, Gal-3 and TAPSE are significantly associated with a positive structural response to CRT.

## 1. Introduction

Cardiac resynchronization therapy (CRT) is an established treatment for heart failure with reduced ejection fraction (HFrEF): randomized controlled trials demonstrate that CRT significantly reduces mortality and hospitalizations in symptomatic patients with left bundle branch block (LBBB) and severe left ventricular (LV) dysfunction [1,2,3]. Despite large application and recognized echocardiographic and ECG cutoffs, many patients are classified as “no-responders”.

Therefore, there is a growing need to identify additional parameters that can help distinguish and select patients most likely to benefit in terms of clinical and functional improvement. In this context, several studies have proposed algorithms incorporating imaging, hemodynamic, and systemic variables to develop more refined and accurate criteria for defining CRT “response”.

Recent position statements have challenged this concept, emphasizing that the expectation of a poor response should not discourage clinicians from implanting a device with proven prognostic benefit. While the degree of left ventricular remodelling may vary among CRT recipients, selecting patients with a higher likelihood of recovery may help optimize resource utilization and clinical management [4].

Among biomarker factors that may impair the efficiency of resynchronization, myocardial inflammation and fibrosis have been described to affect LV adverse remodelling. Soluble suppression of tumorigenicity 2 (sST2), galectin-3 (Gal-3) and the N-terminal portion of the B-type natriuretic peptide (NT-proBNP) have been related to cellular death, myocardial fibrosis, inflammation and congestion [5,6,7]. However, studies examining the relation between circulating levels of those biomarkers, cardiac fibrosis and reverse cardiac remodelling showed conflicting results [8,9]. Our group recently showed that, in a population of HFrEF implanted with CRT, a combined evaluation of soluble suppression of sST2, Gal-3 and an estimate of renal function correlated with cardiovascular outcomes; conversely, the echocardiographic definition of CRT response did not [10].

In this paper, we aim to evaluate the correlation and the dynamic changes of such biomarkers of fibrosis, inflammation, congestion and renal function with echocardiographic data and the structural response to CRT.

## 2. Methods

We retrospectively evaluated consecutive patients undergoing implantation of CRT pacing (CRT-P) or CRT and defibrillation (CRT-D) in our institution, “Azienda Ospedaliera-Universitaria Careggi”, from January 2017 to December 2019. Criteria for CRT candidacy, left bundle branch block definition, exclusion criteria, type and technique of CRT implantation and programming have been previously described. In summary, patients were enrolled if affected by symptomatic HFrEF despite optimal medical therapy, LV ejection fraction ≤ 35% and LBBB morphology with QRS width ≥ 130 ms.

sST2, Gal-3, NT-proBNP, creatinine and estimated glomerular filtration rate (eGFR) with Chronic Kidney Disease Epidemiology Collaboration (CKD-EPI) equation were measured at baseline and after 12 months of CRT implantation. Δ represents the difference between biomarkers at baseline and follow-up. All patients underwent a cardiologic evaluation and echocardiographic study before CRT implantation and 1 1-year follow-up. Responder status was defined as the reduction of LV end-systolic volume ≥ 15% at 1-year follow-up. We also performed a secondary analysis considering responder status as improvement in LVEF ≥ 10% at 1-year follow-up. Our study is in accordance with the ethical guidelines of the 1975 Declaration of Helsinki and was approved by our local institutional review board. Informed consents were obtained from all the patients.

### 2.1. Biomarkers

All blood samples obtained from the patients were collected with a sterile disposable syringe containing EDTA. They were analyzed using the Alere Triage BNP Test. This test is an immunoassay in a single-use plastic cartridge containing a monoclonal antibody for BNP, labeled with a fluorescent dye and BNP. Plasma BNP was measured with the Triage BNP Test (Biosite Inc., San Diego, CA, USA). The human galectin-3 ELISA is an enzyme-linked immunosorbent assay for the quantitative detection of human galectin-3 (Platinum Elisa, eBioscience, San Diego, CA, USA). The assay was performed by measuring the protein in EDTA plasma. Aliquots of serum samples were stored at temperatures ranging from 2° to 8°, and the human galectin-3 levels were determined after 24 h. Each sample was manually measured, and it was assayed in duplicate; a calibration curve was built making serial dilution, starting from a value of 25,000 ng/mL to a value of 0.39 ng/mL. The final reading was realized using a specific scanner (DV 990 BV 4/6, N.T. laboratory, Rome, Italy). The Presage sST2 assay is a quantitative sandwich monoclonal ELISA in a 96-well microtiter plate format for the measurement of sST2 in serum, EDTA plasma, or heparin plasma. The Presage sST2 assay utilizes two mAbs against ST2. A mouse monoclonal antihuman sST2 antibody is coated onto the surface of the microtiter plate wells and acts as the capture antibody to bind sST2 molecules in the solution. A second mouse monoclonal antihuman sST2 antibody is provided in the solution and functions as the tracer antibody for detecting ST2 molecules that bind to the capture antibody (Critical Diagnostics, San Diego, CA, USA).

### 2.2. Echocardiography

All patients were evaluated with a cardiologic visit and echocardiographic study at baseline, before CRT implantation, and at 1-year follow-up. The echocardiographic evaluation was interpreted and independently reviewed by three senior cardiologists according to the instructions provided by the American Society of Echocardiography [11]. Echocardiographic evaluations were performed using GE Vivid E95 systems. The LV volumes and LVEF were measured using the apical two- and four-chamber views by the Simpson biplane formula. Left atrial (LA) dimensions were assessed by measurement of the LA area. The pulsed-Doppler (PW) transmitral flow velocity was used to obtain the early diastolic velocity (E wave), late diastolic velocity (A wave), and their ratio (E/A), and the deceleration time of the E wave. Tissue Doppler peak early diastolic wave (e’) was derived from the apical 4-chamber view at the basal level of the septal and lateral wall and was used to calculate the E/e’ ratio. Right ventricular (RV) longitudinal systolic function was assessed by measuring tricuspid annular systole excursion (TAPSE) by M-mode. The delta (Δ) was defined as the difference between the echocardiographic data (LV volumes, LVEF) at baseline and 1-year follow-up.

### 2.3. Statistical Analysis

Continuous variables, reported as mean ± standard deviation (SD) or as median and interquartile range, were compared between responders and non-responders with Student’s *t*-test or non-parametric tests. Non-continuous variables are expressed with the median and interquartile range. The χ^2^ or Fisher exact test was used to compare non-continuous variables expressed as proportions. Categorical variables reported as percentages were compared between groups with the chi-squared test (or a Fisher’s exact test when any expected cell count was <5).

The predictive parameters of response to CRT were determined by receiver operating characteristic (ROC) curves to identify the best cut-off values. Cox regression model was used to assess the parameters associated with response to CRT. Given the relatively low numbers of patients, we built different models, including at least one clinical, one echocardiographic, and one laboratory parameter. The ones with the highest log-likelihood were then selected. *p*-values are considered significant when <0.05. All analyses were performed using IBM SPSS Statistics for Macintosh, Version 26.0.

## 3. Results

### 3.1. Baseline Characteristics

A total of 86 patients fulfilled the inclusion criteria and were enrolled in the study. Mean age was 70 ± 9 years, mean QRS duration 165 ± 21 ms, LVEF 26% [22;31] and 43% of them had ischemic cardiomyopathy.

### 3.2. Baseline Clinical and Echocardiographic Data of Responders vs. Non-Responders

Baseline characteristics of responder vs. non-responder populations are reported in Table 1. A total of 51 (59%) patients met the criteria of LV reverse remodelling at 1 year follow-up and were considered responders to CRT. When dichotomized in terms of CRT response, responders were more frequently female (9% vs. 37%, *p* = 0.004) and had less frequency of ischaemic aetiology (22% vs. 54%, *p* = 0.03), and lower incidence of diabetes (21% vs. 43%, *p* = 0.03). BSA was lower in responder patients than in non-responders, and they were less frequently active or former smokers. Median LVEF was 25% [23;29] in responders vs. 28% [22;32] non-responders without statistically significant difference, while baseline LV volumes were lower in the CRT responder group (LVEDV 197 ± 65 mL vs. 229 ± 65 mL, *p* = 0.03: LVESV 143 ± 53 mL vs. 169 ± 52 mL, *p* = 0.002). Left atrial LA area, E wave and E/A ratio did not differ between non-responder and responder patients. E/e’ was lower in responders compared to non-responders (14 [10;17] vs. 18 [16;21], *p* = 0.001) while TAPSE was higher (20 [17;21] mm vs. 15 [14;30] mm, *p* < 0.001).

### 3.3. Baseline Biomarker Levels

**Figure 1:**
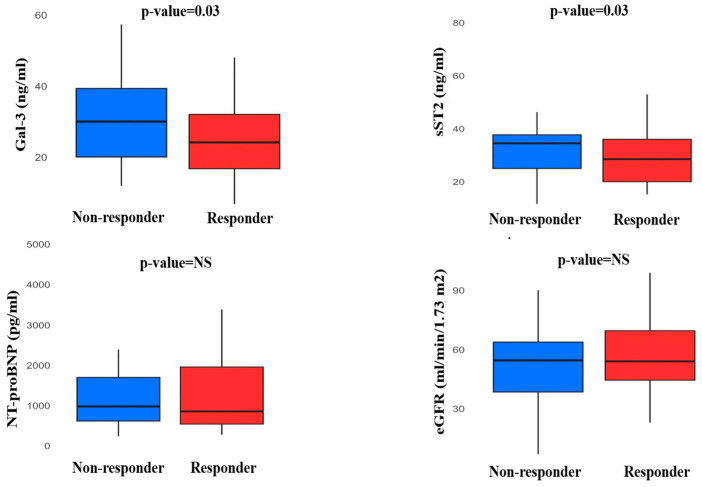
Baseline levels of biomarkers in responders and non-responders to CRT.

Baseline levels of Gal-3 (responders: 24.1 [16.8;32] ng/mL, non-responders: 30 [20;39.3] ng/mL, *p* = 0.03) and sST2 (responders: 28.5 [20;36] ng/mL, non-responders: 34.5 [25;37.7] ng/mL, *p* = 0.03) were observed to be lower in responders than non-responders. Biomarkers of renal function did not significantly differ between responders and non-responders as baseline levels (creatinine 1.1 [1.0;1.5] mg/dL, vs. 1.2 [1.1;1.6] mg/dL, eGFR 54.0 [44.5;69.5] mL/min/1.73 m^2^ vs. 54.5 [38.5;63.75 mL/min/1.73 m^2^, respectively). Moreover, there was no significant difference in NT-proBNP values (responders: 883 [554;2175] pg/mL, non-responders: 1394 [671;2267] pg/mL) (Figure 1). Considered as a continuous measure, baseline Gal-3 levels showed a significant linear relationship with eGFR (r = −0.4, *p* = 0.001) and TAPSE (r = −0.5, *p* = 0.001).

### 3.4. Biomarkers Trend at Follow-Up

**Figure 2:**
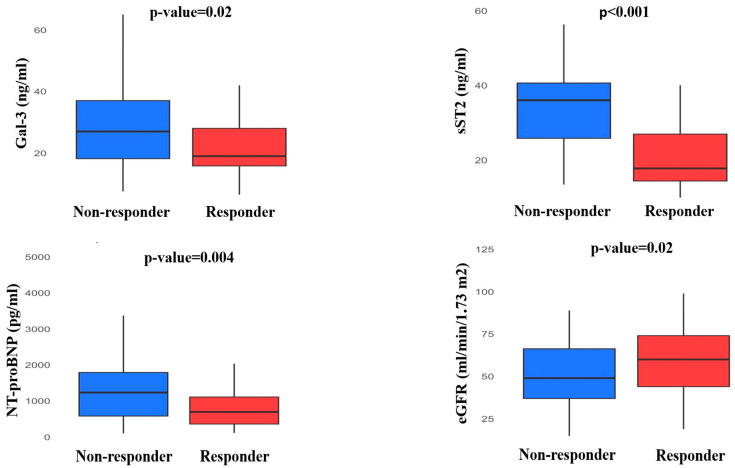
Follow-up levels of biomarker in responders and non-responders to CRT.

Differences in biomarker levels according to CRT response at follow-up are shown in Table 2. Plasma levels of Gal-3, sST2 and NT-proBNP were lower in patients that achieved a positive response to CRT (responders: Gal-3 19 [15.8;28] pg/mL, non-responders: 27 [18.20;37] pg/mL, *p* = 0.02; responders: sST2 17.8 [14.45;26.95] pg/mL, non-responders: 36 [25.85;40.60] pg/mL, *p* < 0.001; responders: NT-proBNP 749 [365;1182] pg/mL, non-responders: 1483 [858;2833] pg/mL, *p* = 0.004). At the 12-month visit, patients responding to CRT showed a significant better estimate of renal function compared to patients that did not respond (responders: eGFR 60 [44;74] mL/min/1.73 m^2^, non-responders 49 [37;66.2] mL/min/1.73 m^2^, *p* = 0.02) (Figure 2). Responders showed a significant reduction between baseline and follow-up values of ΔGal-3 (−12.1 [−23.4;3.5]% vs. −2.5 [−19.2;2.3]%, *p* = 0.04), ΔsST2 (−30.8 [−35.8;−20.2]% vs. 2.2 [−0.5;4.9]%, *p* < 0.001), ΔNT-proBNP (−16.4 [−48.1;25.5]% vs. 5.2 [−27.5;53.3], *p* = 0.04) and ΔeGFR (6.7 ± 24.3% vs. −6.3 ± 27.9%, *p* = 0.03).

### 3.5. Prediction of Response to Cardiac Resynchronization Therapy

Receiving operating characteristic (ROC) curve analysis was performed to define the optimal cut-off value for biomarkers and echocardiographic data to predict the response to CRT. The cut-off values with the highest accuracy were 38.5 pg/mL for Gal-3 (AUC = 0.63 [0.51;0.75], *p* = 0.03), 23.3 pg/mL for sST2 (AUC = 0.64 [0.51;0.77], *p* = 0.03), 15.5 for E/e’ (0.70 [0.59;0.82], *p* = 0.001) and 17.5 mm for TAPSE (AUC = 0.72 [0.61;0.84], *p* < 0.001).

Multivariate logistic regression analyses were performed with the identified cut-offs of laboratory values and echocardiographic data, together with the most relevant clinical parameters. Several predictive models were built; we report the ones achieving the highest log-likelihood. Gal-3 ≤ 38.5 pg/mL confirmed as an independent predictive parameter of echocardiographic CRT response (OR 7.13 [1.12;45.41], *p* = 0.03), together with TAPSE > 17.5 mm (OR 10.86 [3.15;37.44], *p* < 0.001) including in the model LVEF (OR 1.01 [0.90;1.12], *p* = 0.83), E/e’ ≤ 15.5 (OR 1.98 [0.57;6.79], *p* = 0.27) and ischemic aetiology (OR 0.45 [0.14;1.47], *p* = 0.18) (Table 3). These parameters maintained their significance in the model, including female sex as a clinical parameter (Table 4). Conversely, the multivariate logistic regression analysis including TAPSE, LVEF, E/e’ ≤ 15.5 and ischemic aetiology together with sST2 did not confirm the role of sST2 as an independent predictive factor of positive response to CRT (sST2 ≤ 23.3 OR 2.30 [0.58;9.09], *p* = 0.23; TAPSE > 17.5 OR 11.21 [3.39;37.04], *p* ≤ 0.001). Moreover, the same model including ΔeGFR showed that the improvement of renal function (OR 1.06 [1.01;1.11], *p* = 0.01), TAPSE (OR 16.06 [3.84;67.09], *p* < 0.001) and sex female (OR 5.91 [1.08;32.34], *p* = 0.04) were independent factors associated with a positive response to CRT (Table 5).

### 3.6. Biomarkers Profile Considering Responder Status as Improvement in LVEF ≥ 10%

When defining ‘responder’ status as an improvement in LVEF ≥ 10% at 1-year follow-up, baseline levels of Gal-3 (responders: 21.5 [16.5–30] ng/mL; non-responders: 32 [20.1–38.2] ng/mL; *p* = 0.001) and sST2 (responders: 26.5 [20–34.7] ng/mL; non-responders: 35.2 [25–37.4] ng/mL; *p* = 0.01) were significantly lower in responders. Responders also showed better baseline renal function, as reflected by eGFR (responders: 57.0 [47.5–73.7] mL/min/1.73 m²; non-responders: 53 [35.5–63] mL/min/1.73 m²; *p* = 0.03). Baseline NT-proBNP levels did not differ significantly between groups. At follow-up, responders demonstrated greater reductions in Gal-3 (ΔGal-3: −13.5 [–23; 3.5]% vs. −2.6 [−20.5; 2.1]%, *p* = 0.03) and sST2 (ΔsST2: −28.7 [−35.4; −20]% vs. 0.9 [−11.6; 2.8]%, *p* < 0.001), as well as a significant improvement in renal function (ΔeGFR: 2.9 ± 11.7% vs. −3.3 ± 14.7%, *p* = 0.03).

Gal-3 ≤ 38.5 pg/mL confirmed its independent predictive role of echocardiographic CRT response (OR 9.77 [1.04;91.83], *p* = 0.03), together with TAPSE > 17.5 mm (OR 8.14 [2.40;27.57], *p* < 0.001) including in the model LVEF (OR 0.98 [0.89;1.08], *p* = 0.75), E/e’ ≤ 15.5 (OR 1.25 [0.30;3.86], *p* = 0.69) and ischemic etiology (OR 0.67 [0.23;1.93], *p* = 0.46) (Supplementary Table S1). These parameters maintained their significance in the model, including female sex or diabetes mellitus as a clinical parameter. Conversely, the multivariate logistic regression analysis including sST2, ischemic etiology and other echocardiographic parameters as TAPSE, LVEF and E/e’ ≤ 15.5 did not confirm the role of sST2 as an independent predictive factor of positive response to CRT.

## 4. Discussion

### 4.1. Cardiac Biomarkers Associated with CRT Response

In this study, we observed in several multivariate prediction models that baseline cut-off values of Gal-3 ≤ 38.5 pg/mL and TAPSE > 17.5 mm were able to identify patients with LV reverse remodelling at 1-year follow-up. Baseline sST2 values, although different among responders and non-responders, did not prove significant at the multivariable prediction models built, along with NT-proBNP. Concerning follow-up values, we found that 1 year after CRT implantation, patients experienced a substantial reduction of Gal-3, sST2, eGFR and NT-proBNP, more pronounced in those with a structural response to CRT. These results corroborate the concept that levels of extracellular matrix and collagen biomarkers are associated with cardiac fibrosis and LV remodelling in HFrEF and can be modulated by HF therapy: serial measurements provide incremental information to baseline levels, reflecting changes in myocardial remodelling over time [12]. Since biomarkers of fibrosis, congestion and inflammation reflect processes involved in reverse cardiac remodelling and carry negative prognostic implications in heart failure, several studies have explored their role—particularly collagen synthesis markers—in the context of cardiac remodelling and CRT outcomes. However, the literature has yielded inconsistent results, likely due to heterogeneous definitions of CRT response, small sample sizes and variability in study design and patient selection—factors often associated with poorer CRT outcomes [13,14]. CARE-HF trial demonstrated that Gal-3 was an independent predictor of death from any cause or unplanned hospitalization for a major cardiovascular event; however, Gal-3 did not predict CRT response when considered as a separate outcome. The different inclusion criteria of the trial (different HF stage, QRS with all morphology and criteria of dyssynchrony for patients with a QRS interval of 120 to 149 ms) may explain this discrepancy from our results [15]. Indeed, several studies demonstrated that Gal-3 is associated with left ventricular remodelling; patients in whom the LVEDV decreased over time had significantly lower levels of Gal-3 [16,17,18]. sST2 is expressed in isolated cardiac myocytes that are exposed to mechanical strain and is widely considered reflective of cardiac remodelling [19]. Levels of sST2 are favourably influenced by currently recommended HFrEF therapy [20]. In a subpopulation of MADIT-CRT, patients with lower baseline sST2 showed a lower risk of ventricular arrhythmias and death than those with higher levels, even when adjusting for baseline BNP, and every 10% increase in sST2 values at 1 year, there was an increased risk of death or VA. However, also in MADIT-CRT, the results did not show a significant correlation between baseline sST2 and LV contractility (assessed by longitudinal strain) [21]. Accordingly, in our study, although baseline sST2 levels did not predict a structural response to CRT, serial measurements provided incremental information to baseline levels, reflecting changes in myocardial remodelling over time [22].

Interestingly, our study did not identify NT-proBNP as a specific predictor of CRT response, although follow-up data showed a more pronounced reduction in NT-proBNP levels among patients with left ventricular reverse remodelling. While we cannot demonstrate a direct relationship with myocardial fibrosis, we speculate that Gal-3—serving as a surrogate marker of collagen burden—may be a more sensitive indicator of reduced cardiac compliance than natriuretic peptides, and could provide complementary prognostic value in CRT candidates [23,24].

Although ischemic etiology was associated with lower CRT response rates in univariate analysis, it lost statistical significance in multivariate models, likely due to the heterogeneity of ischemic patients, including those with limited scar burden or early revascularization. Conversely, galectin-3 remained independently associated with reverse remodeling across multiple models, suggesting that the extent of myocardial fibrosis, rather than the underlying etiology itself, may better reflect the myocardial substrate amenable to reverse remodeling. Similarly, TAPSE preserved its predictive value, suggesting that right ventricular dysfunction may also influence the echocardiographic response to CRT rather than ischemic etiology and should be considered in multiparametric risk stratification. CRT has been shown to improve reverse remodelling across all stages of CKD, although to a lesser extent in patients with advanced renal dysfunction (CKD stage 3–5). However, patients with CKD derive a benefit on outcome at a lesser degree of remodelling [25]. A reduction in the slope of eGFR is an effect of CRT-induced LV reverse remodelling and predicts better survival in patients with moderately reduced eGFR, rather than the absolute baseline values [26]. Accordingly, in our analysis, CRT improved renal function independently from baseline eGFR, and patients who did not respond to CRT showed a significant decline of eGFR at follow-up compared to responders. Moreover, we found a significant linear relationship between Gal-3 and eGFR that may be explained by the correlation between fibrosis and renal function: Gal-3 is involved in both the cardiac and renal fibrogenesis, leading to cardiac dysfunction or kidney injury in several humans and experimental models of HF and kidney disease [27].

### 4.2. Echocardiographic Predictors of CRT Response

In our cohort, indirect signs of baseline lower filling pressures were observed in patients with a favourable response to CRT, confirming that the effects of CRT on LV diastolic function are also dependent on the PW-determined filling characteristics before device implant [28]. These findings align with the pathophysiological rationale that increased myocardial stiffness—resulting from greater fibrosis and reflected by elevated E/e’ ratios—is associated with a less favorable response to CRT [29]. As already demonstrated in the REVERSE trial, our data confirm that TAPSE, a widely recognized and feasible marker of RV systolic function, is an independent predictor of the echocardiographic response to CRT; a cut-off of 17.5 mm showed good AUC at the ROC analysis and proved significant at several multivariate prediction models [30]. Accordingly, the CARE-HF trial demonstrated that the response to CRT in patients with severely reduced TAPSE was smaller than in patients with preserved TAPSE [31]. Previous studies have not found relationships between TAPSE and interventricular mechanical delay or QRS duration, indicating that TAPSE is not a marker of LV dyssynchrony [32], but it predicts outcome in patients with symptomatic HFrEF as a surrogate of RV function, independently from LVEF and pulmonary hypertension [33].

Bragança et al. confirmed that the TAPSE/PASP ratio, reflecting the coupling between right ventricular longitudinal function and pulmonary pressure, is a well-established prognostic marker capable of predicting response to CRT [34]. Moreover, the RV dysfunction in HF patients may result from several pathological mechanisms, including RV remodelling and fibrosis. Recent studies have described potential mechanisms linking Gal-3 to right ventricular function and clinical outcomes in heart failure populations, with growing evidence supporting a connection between myocardial fibrosis and impaired right heart function [35]. In fact, myocardial fibrosis in a failing heart might be considered as part of an adaptive response to prevent cardiomyocyte overstretch and to maintain RV shape for contraction, which, however, increases diastolic stiffness, perturbs cardiomyocyte excitation–contraction coupling, and leads to an abnormal myocardial contraction [36].

### 4.3. Researching Predictors of CRT Response: Still a Role in Clinical Practice?

The “response” to CRT is a traditional concept derived from the attempt to identify how CRT works in terms of clinical improvement and reverse remodelling. Indeed, this old-fashioned definition has been recently mostly abandoned, and clinical stabilization is now considered an equally important endpoint. In a recent paper, we showed that biomarkers such as Gal-3 and sST2 were predictors of clinical outcomes after CRT implantation, as long as E/e’, in line with the cited literature. However, in the analysis, the echocardiographic definition of response to CRT was not related to the same outcome. Most cited studies did not find a correlation of these biomarkers with the echocardiographic CRT “response”, but only with outcomes. This seems to corroborate the hypothesis that the response to CRT and clinical outcomes are somehow independent elements that cannot necessarily be correlated.

Conversely, in line with the ESC position paper mentioned above, we agree that patients who meet guideline-based criteria for CRT should undergo implantation without delay. Our study provides food for thought for those with borderline criteria (e.g., QRS between 130 and 150 ms) where the recommendation class is weaker: in these patients, a multiparametric evaluation is useful to add information and potentially support the indication for implantation. Furthermore, even in patients with strong CRT criteria, disposing of data that predict a poor echocardiographic response can be an element to anticipate follow-up and implement earlier strategies for HF.

## 5. Limitations

As already extensively acknowledged, all data were collected retrospectively from our single centre, allowing the achievement of a small sample size. SGLT-2 inhibitors were not prescribed because not available at the time of CRT implantation. However, we included an accurately screened population undergoing CRT implantation, following the indication of the current guidelines. A major limitation of the study is the exclusion of patients with a QRS > 150 msec and a non-LBBB morphology; however, CRT response is more consistent and predictable in those with LBBB with the highest level of evidence and strongest guideline support.

The lack of a centralized and independent echocardiographic core lab for image assessment represents a limitation in interpreting our findings, given that the study hinges on the response to CRT in terms of cardiac volume reduction. The “response” to CRT depends on several parameters: underlying aetiology, loss of LV capture, percentage of biventricular pacing and compliance to pharmacological treatment cannot be ruled out. TAPSE is a commonly used and easily measured RV function parameter, but it only evaluates the longitudinal movement of the basal segment of the RV free wall and may fail to accurately reflect the entire RV performance. Moreover, Gal-3 and sST2 levels may be influenced by chronic inflammatory diseases and/or renal failure. We did not have data regarding other inflammatory biomarkers (e.g., cytokines, interleukins, tumor markers, Erythrocyte Sedimentation Rate Test and that could provide further mechanistic insight into inflammation and CRT response.

## 6. Conclusions

In a selected population of HFrEF patients implanted with CRT, laboratory values, including baseline Gal-3, together with TAPSE, were correlated to the structural response to CRT. The search progression for parameters of CRT response might still have a role in clinical practice, mainly to support implantation. Despite the potential confounders, our results encourage clinicians to serially assess the levels of cardiac biomarkers of fibrosis and renal function that provide significant pathophysiological and practical information in HFrEF patients before proceeding with CRT implantation.

## Figures and Tables

**Table 1:** Baseline clinical, biomarkers and echocardiographic features according to cardiac resynchronization therapy response.

	Non-Responder *n* = 35	Responder *n* = 51	*p*
Age	71 ± 7	68 ± 10	NS
Sex female (*n*,%)	3(9)	19(37)	0.004
BSA (mq)	1.9 ± 0.1	1.8 ± 0.2	0.01
Ischaemic aetiology	19 (54)	18 (22)	0.03
Diabetes	15 (43)	11 (21)	0.03
Smoke active	10 (28)	13 (25)	0.003
Former	11 (31)	6 (12)	0.003
Arterial hypertension	20 (57)	25 (49)	NS
Dyslipidaemia	19 (54)	22 (43)	NS
Atrial fibrillation	15 (42)	6 (11)	0.004
NYHA class			
II (*n*,%)	10 (28)	13 (25)	NS
III (*n*,%)	19 (54)	34 (67)	NS
IV (*n*,%)	6 (17)	4 (8)	NS
*Biomarkers*			
Creatinine (mg/dL)	1.2 [1.1;1,6]	1.1 [1.0;1.5]	NS
eGFR (mL/min/1.73 m^2^)	54.5 [38.5;63.7]	54.0 [44.5;69.5]	NS
NT-proBNP (pg/mL)	1394 [671;2267]	883 [554;2175]	NS
Gal-3 (ng/mL)	30 [20;39.3]	24.1 [16.8;32]	0.03
sST2 (ng/mL)	34.5 [25;37.7]	28.5 [20;36]	0.03
Echocardiographic data			
LVEDV (mL)	229 ± 65	197 ± 65	0.03
LVESV (mL)	169 ± 52	143 ± 53	0.02
LVEF (%)	25 [23;29]	28 [22;32]	NS
LVDD (mm)	71 ± 9	64 ± 8	0.001
LVDS (mm)	59 ± 9	51 ± 9	<0.001
E wave (cm/sec)	85 ± 28	80 ± 24	NS
E/A	1.2 [0.7;1,7]	0.9 [0.6;1.8]	NS
E/e’	18 [16;21]	14 [10;17]	0.001
LA area (cm^2^)	24 [21;27.5]	23 [19;27]	NS
TAPSE (mm)	15 [14;20]	20 [17;21]	<0.001
Treatment			
B-blockers (*n*,%)	32 (92)	47 (94)	NS
ACEi/sacubitril–valsartan (*n*,%)	30 (86)	45 (88)	NS
MRA (*n*,%)	28 (82)	43 (84)	NS
Diuretic (*n*,%)	33 (95)	48 (95)	NS

Baseline clinical, biomarker, echocardiographic and pharmacologic data. All values are expressed as absolute numbers (*n*) and (%) for categorical variables or mean ± standard deviation for parametric or median [first quartile; third quartile] for non-parametric continuous variables. Angiotensin-converting-enzyme inhibitors (ACEi), body surface area (BSA), estimated glomerular filtration rate (eGFR), galectin-3 (Gal-3), New York Heart Association (NYHA), left ventricular end diastolic volume (LVEDV), left ventricular end systolic volume (LVESV), left ventricular ejection fraction (LVEF), left ventricular diastolic diameter (LVDD), left ventricular systolic diameter (LVDS), left atrium (LA), mineralcorticoid receptor antagonist (MRA), tricuspid anular post systolic excursion (TAPSE), not significant (NS).

**Table 2:** Biomarkers and echocardiographic features according to cardiac resynchronization therapy response at follow-up.

	Non-Responder *n* = 35	Responder *n* = 51	*p*
*Biomarkers*			
Creatinine (mg/dL)	1.57 ± 0.63	1.25 ± 0.45	0.008
eGFR (mL/min/1.73 m^2^)	49 [37;66.2]	60 [44;74]	0.02
ΔeGFR (%)	−6.3 ± 27.9	6.7 ± 24.3	0.03
Gal-3 (ng/mL)	27 [18.2;37]	19 [15.8;28]	0.02
ΔGal-3 (%)	−2.5 [−19.2;2.3]	−12.1 [−23.4;3.5]	0.04
sST-2 (ng/mL)	36 [25.8;40.6]	17.8 [14.4;26.9]	<0.001
ΔsST-2 (%)	2.2 [−0.5;4.9]	−30.8 [−35.8;−20.2]	<0.001
NT-proBNP (pg/mL)	1483 [858;2833]	749 [365;1182]	0.004
ΔNT-proBNP (%)	5.2 [−27.5;53.3]	−16.4 [−48.1;25.5]	0.04
Echocardiographic data			
LVEDV (mL)	239 ± 72	140 ± 50	<0.001
LVESV (mL)	175 ± 58	81 ± 40	<0.001
LVEF (%)	26 [24;41.5]	45 [38;50]	<0.001
LVDD (mm)	72 ± 9	59 ± 8	<0.001
LVDS (mm)	60 [53;65]	43 [39;52]	<0.001
E wave (cm/sec)	87 ± 28	69 ± 25	0.004
E/A	1.2 [0.6;2.0]	0.77 [0.60;1.1]	0.04
E/e’	18 [12.7;22.7]	11 [9.0;15.2]	<0.001
E/e’ < 15	8(22)	31(60)	0.001
LA area (cm^2^)	27 ± 6	24 ± 6	0.01
TAPSE (mm)	14.5 [14;16.25]	20 [18;22]	<0.001
TAPSE > 17.5	9 (25)	43 (84)	<0.001

Follow-up clinical, biomarker, echocardiographic and pharmacologic data and delta values between baseline and follow-up. All values are expressed as absolute numbers (*n*) and (%) for categorical variables and mean ± standard deviation for parametric or median [first quartile; third quartile] for non-parametric continuous variables. Body surface area (BSA), estimated glomerular filtration rate (eGFR), galectin-3 (Gal-3), New York Heart Association (NYHA), left ventricular end diastolic volume (LVEDV), left ventricular end systolic volume (LVESV), left ventricular ejection fraction (LVEF), left ventricular diastolic diameter (LVDD), left ventricular systolic diameter (LVDS), left atrium (LA), tricuspid anular post systolic excursion (TAPSE).

**Table 3:** Prediction model with multivariable logistic regression analysis considering Gal-3, significant echocardiographic parameters and ischemic etiology for favourable response to cardiac resynchronization therapy.

	Odds Ratio [CI 95%]	*p*-Value	Log Likelihood = −37
Biomarkers			
Galectin-3 (pg/mL) ≤ 38.5	7.13 [1.12;45.41]	0.03	
E/e’ ≤ 15.5	1.98 [0.57;6.79]	0.27	
TAPSE (mm) > 17.5	10.86 [3.15;37.44]	<0.001	
LVEF (%)	1.01 [0.90;1.12]	0.83	
Ischemic etiology	0.45 [0.14;1.47]	0.18	

Left ventricular ejection fraction (LVEF), tricuspid anular post systolic excursion (TAPSE).

**Table 4:** Prediction model with multivariable logistic regression analysis considering Gal-3, significant echocardiographic parameters and female sex for favourable response to cardiac resynchronization therapy.

	Odds Ratio [CI 95%]	*p*-Value	Log Likelihood = −35
Biomarkers			
Galectin-3 (pg/mL) ≤ 38.5	10.51 [1.42;77.73]	0.02	
E/e’ ≤ 15.5	1.82 [0.49;6.77]	0.36	
TAPSE (mm) > 17.5	8.91 [2.42;32.81]	0.001	
LVEF (%)	1.0 [0.89;1.11]	0.83	
Sex (female)	6.21 [0.97;39.63]	0.05	

Left ventricular ejection fraction (LVEF), Tricuspid anular post systolic excursion (TAPSE).

**Table 5:** Prediction model with multivariable logistic regression analysis considering eGFR, significant echocardiographic parameters and female sex for favourable response to cardiac resynchronization therapy.

	Odds Ratio [CI 95%]	*p*-Value	Log Likelihood = −33
Biomarkers			
ΔeGFR (ml/min/1.73 m^2^)	1.06 [1.01;1.11]	0.01	
E/e’ ≤ 15.5	1.41 [0.35;6.34]	0.62	
TAPSE (mm) > 17.5	16.06 [3.84;67.09]	<0.001	
LVEF (%)	0.96 [0.85;1.09]	0.56	
Sex (female)	5.91 [1.08;32.34]	0.04	

Multivariate logistic regression analysis considering eGFR calculated with CKD-EPI formula, significant echocardiographic and clinical baseline predictive parameters and LVEF. Estimated glomerual filtration rate (eGFR), left ventricular ejection fraction (LVEF), tricuspid anular post systolic excursion (TAPSE).

## Data Availability

The datasets presented in this article are not readily available due to time limitation. Requests to access the datasets should be directed to the authors.

## Supplementary Materials

The following supporting information can be downloaded at https://www.mdpi.com/article/10.3390/jcm14103496/s1, Table S1: Multivariate logistic regression analysis.
