# Supplementary material for: Combination Between Biomarkers and Echocardiographic Data for Prediction of Left Ventricular Reverse Remodelling in Cardiac Resynchronization Therapy
Source: J Clin Med. 2025 May 16;14(10):3496. doi: 10.3390/jcm14103496 (PMC12112598; doi:10.3390/jcm14103496)
Supplement: Supplementary file 1 [file jcm-14-03496-s001.zip › jcm-3553259-supplementary.pdf]

Table S1. Multivariate logistic regression analysis.

	**Odds ratio[CI 95%]**	**P-value**	**Log likelihood=37,5**
Biomarkers			
**Galectin-3 (pg/ml) ≤ 38,5**	**9.77 [1.04;91.83]**	**0.03**	
E/e′ ≤15,5	**1.25 [0.30;3.86]**	**0.69**	
**TAPSE (mm) > 17,5**	**8.14 [2.40;27.57]**	**<0.001**	
**LVEF (%)**	**0.98 [0.89;1.08]**	**0.75**	
**Ischemic aetiology**	**0.67 [0.23;1.93]**	**0.46**	

Multivariate logistic regression analysis considering as responder patient with 10% improvement in LVEF. Significant baseline predictive parameters and LVEF were examined. Left ventricular ejection fraction (LVEF), Tricuspid anular post systolic excursion (TAPSE).
